# Common carotid pulsatility is deteriorated by autoimmune thyroiditis in children with type 1 diabetes mellitus – A pilot study

**DOI:** 10.14814/phy2.14518

**Published:** 2020-08-03

**Authors:** Jolanta Neubauer‐Geryk, Melanie Wielicka, Grzegorz Kozera, Małgorzata Myśliwiec, Katarzyna Zorena, Leszek Bieniaszewski

**Affiliations:** ^1^ Clinical Physiology Unit Medical Simulation Centre Medical University of Gdańsk Gdansk Poland; ^2^ Department of Pediatrics, Diabetology and Endocrinology Medical University of Gdańsk Gdansk Poland; ^3^ Department of Immunobiology and Environmental Microbiology Medical University of Gdańsk Gdansk Poland

**Keywords:** autoimmune thyroiditis, carotid pulsatility index, children, type 1 diabetes mellitus, vascular aging

## Abstract

Autoimmune thyroiditis (AIT) frequently coexists with type 1 diabetes (DM1) and additionally increases the extent of microcirculatory complications due to DM1. We hypothesized that in pediatric patients with DM1, impairment of macrocirculation could be further augmented by a coexisting autoimmune process. Therefore, we investigated the influence of AIT on large arteries in DM1 pediatric patients. Our group consisted of 19 DM1, 19 DM1 + AIT patients and 29 control subjects. The groups were comparable regarding age and gender. The DM1 and DM1 + AIT patients were matched for age at onset of DM1 and diabetes duration. Macrocirculation was described using pulsatility indices (PIs) determined for common carotid (CCA) and peripheral arteries of upper and lower limbs. CCA resistance index (RI) and ABI were also assessed. Children with DM1 + AIT had only significantly lower CCA_PI and CCA_RI in comparison with controls whereas in the absence of AIT such difference was not found. The diabetes duration and age of onset did not correlate with carotid indices. Total cholesterol level was higher both in DM1 + AIT and DM1 groups than in the control group. For low density lipoproteins cholesterol, a significant difference was found between DM1 + AIT and control groups. Age‐independent impact of AIT on CCA_PI was confirmed by multivariate analysis. Common carotid pulsatility is deteriorated by autoimmune thyroiditis independently of age in children with type 1 diabetes mellitus.

## Graphical abstract

Autoimmune thyroiditis frequently coexist with type 1 diabetes and additionally increases the extent of microcirculatory complications due to type 1 diabetes mellitus, but its impact on large arteries has been rarely studied. We investigated the influence of autoimmune thyroiditis on large arteries in type 1 diabetes mellitus pediatric patients. It was concluded that common carotid pulsatility is deteriorated by autoimmune thyroiditis independently of age in children with type 1 diabetes mellitus.

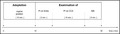

## 1 INTRODUCTION

Type 1 diabetes mellitus (DM1) is the second most common chronic childhood disease. Significant improvements in management and survival have been observed over the last century. However, mortality from type 1 diabetes is still increased two to eightfold when compared to general population (Lind et al., 2014; Rawshani et al., 2017), which reflects a loss of about 12 years of life expectancy at age 20. Cardiovascular disease, with its earlier onset and increased severity, is still the main cause of morbidity and mortality in adults with type 1 diabetes. Therefore, aggressive management of cardiovascular risk factors in type 1 diabetes, especially after the age of 40 or with symptoms of microvascular complications is recommended. Recently, similar recommendations appeared for the pediatric population. Furthermore, in younger DM1 patients the coexistence of autoimmune thyroid disease (DM1 + AIT) aggravates the deterioration of microcirculation function (Hoffmann et al., 2019). We hypothesized that in pediatric patients with DM1, impairment of macrocirculation could be further augmented by a coexisting autoimmune processes. Therefore, we decided to examine the characteristics of flow in large arteries, both elastic and muscular, in pediatric patients with DM1 and autoimmune thyroiditis.

## 2 METHODS

### 2.1 Study groups

For the study we recruited patients with type 1 diabetes who are under long‐term care of the Department of Pediatrics, Diabetology and Endocrinology, University Clinical Center in Gdansk. The research was carried out from July 2014 to 2018 at the Physiology Unit of Medical Simulation Center, Medical University of Gdansk after obtaining referrals from the doctors responsible for their long‐term treatment. The control group was recruited out of the age‐matched volunteers.

The study group consisted of 19 pediatric patients with type 1 diabetes, 19 subjects with type 1 diabetes and coexisting autoimmune thyroiditis matched for age, gender, age at onset of DM and diabetes duration and 29 healthy subjects of similar gender, age and BMI, who served as control group (Table 1). The only medication used by the patients was insulin, administered either through a pump or a pen. Based on medical history, physical examination and biochemical analysis none of the study subjects had any form microangiopathy, including retinopathy, nephropathy or neuropathy (American Diabetes Association, 2016; Dyck, 1988; Singer, Schachat, & Nathan, 1992). All examinations were performed between 8:00 a.m. and 1:00 p.m. The study protocol consisted of obtaining medical history, determination of pulsatility index (PI) and ankle‐brachial index (ABI) determination and laboratory testing. Patients from all study groups were euthyroid at the time of investigation.

**Table 1:** Characteristics of study groups

Characteristics	Healthy control (C), *N* = 29	Diabetes mellitus t.1 (DM1), *N* = 19	Diabetes mellitus t.1 and autoimmune thyroiditis (DM1 + AIT), *N* = 19	*p*
Male gender (%)	52	42	42	.74
Body mass (kg)	52.8 (29.0–80.1)	54.8 (29.9–78.5)	59.2 (43.5–80.3)	.50
Body mass index (kg/m^2^)	19.1 (14.4–24.7)	19.8 (15.0–27.4)	20.5 (16.1–26.8)	.22
Age (years)	14.7 (11.3–17.7)	16 (10.9–17.8)	15.2 (11.1–18.0)	.20
Age at onset of diabetes (years)	–	9.3 (2.5–13.5)	6.6 (1.2–12.2)	.07
Diabetes duration (years)	–	7 (1.7–12.9)	8.7 (2.6–14.4)	.16
Insulin dose (units/24h)	–	40 (25–100)	47 (20–90)	.30
Treatment with pump (%)	–	53	74	.18
HbA1C (mmol/mol/%)	34 (29–38)/5.3 (4.8–5.6)	60 (44–103)/7.6 (6.2–11.6) *p = .000 versus C	64 (41–123)/8 (5.9–13.4) *p = .000 versus C	**<.001**
TSH (mIU/l)	2.1 (0.7–5.1)	1.5 (0.6–3.1)	1.9 (1–3.9)	.07
fT4 (pmol/L)	12.5 (9.3–15)	12.9 (10.9–15.1)	12.7 (10.1–18.1)	.65
Albuminuria (mg/dl)	–	6 (2.5–27)	10.1 (2.5–20.6)	.69
Creatinine (mg/dl)	0.63 (0.52–0.94)	0.7 (0.5–0.9)	0.6 (0.5–0.9)	.46
eGFR (ml/min)	105 (77–125)	99 (79–147)	107 (77–135)	.24
Total cholesterol (mg/dl)	163 (120–218)	183 (134–248) *p=.048 versus C	180 (129–270) *p=.004 versus C	**.003**
Cholesterol LDL (mg/dl)	94.5 (64–127)	102 (61–138)	102 (68–180) *p=.02 versus C	**.02**
Cholesterol HDL (mg/dl)	50.5 (41–82)	54 (35–90)	60 (38–65)	.11
Triglicerydes (mg/dl)	53.5 (36–117)	70 (37–294)	81 (34–160)	.03
SBP (mmHg)	109 ± 9.3	103 ± 9.1	107 ± 8.4	.09
DBP (mmHg)	62 ± 4.9	58 ± 5.2	61 ± 6.4	.62
HR (beats/minutes)	79 ± 11.8	77 ± 10.2	84 ± 13.4	.13

Note

Values are presented as mean ± *SD* or median (minimum; maximum). The value of *p* < .05 was regarded as statistically significant (in bold).

Abbreviation: HbA1C: glycated hemoglobin; TSH: Thyroid‐stimulating hormone; fT4: free thyroxine; eGFR: estimated glomerular filtration rate; SBP: systolic blood pressure; DBP: diastolic blood pressure; HR: heart rate; LDL, low density lipoproteins.

^a^ The level of significance of comparison between the groups according to post hoc Fisher's test.

The results of arterial examination are stored at the Clinical Physiology Department. To enable sharing out data, patients' identifying information was replaced with observation numbers.

### 2.2 Examination of large arteries

**Figure 1:**
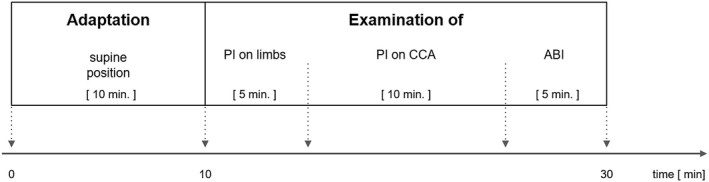
The project timeline

The test was carried out in a quiet room with a constant temperature of about 20°C. Examination were performed after 10 min of adaptation in a supine position and lasted 30 min (Figure 1).

The flow characteristics of the common carotid, brachial and lower limb arteries were studied using the VasoGuard 5000 device (Nicoletes). The cuff was placed on the lower limb in four locations: on the upper part of the thigh, above the knee, below the knee, and over the ankle. The measurements were performed simultaneously on both sides. The device software allows to determine pulsatility index (PI) for all studied arteries, while determination of the resistance index (RI) was restricted to common carotid arteries only. Indices were calculated according to Gosling (PI) and Pourcelot (RI) formulas.

PI=(peak systolic velocity‐minimal diastolic velocity)/mean velocity,

$$\text{RI}=\left(\text{peak systolic velocity}‐\text{minimal diastolic velocity}\right)/\text{peak systolic velocity}.$$

The mean values of indices from both sides were used for further testing. The measurements at the common carotid artery were taken three times on each side, which was followed by calculation of the mean pulsatility index and resistance in accordance with the specified formulas.

The ankle‐brachial index (ABI) was determined automatically using the VasoGuard (Nicoletes) device for both lower limbs at the same time.

### 2.3 Statistical analysis

All the analyses were performed using STATISTICA data analysis software system, version 12. (StatSoft, Inc., Tulsa, OK, USA). Shapiro–Wilk tests were performed to analyze the distribution of continuous variables. Values are expressed either as median and range or as mean and standard deviation as appropriate. For group changes, Wilcoxon test was used. The comparison between groups was performed using the Mann–Whitney test. The chi‐squared test was used to compare proportions between genders. The changes between groups in pulsatility index at the carotid and brachial artery, as well as the lower leg arteries at the ankle level were analyzed using MANOVA. The comparison between groups with regards to the associated continuous variables was performed using General Linear Model with Fisher test for post hoc analysis when needed. An annotation indicating the use of post hoc data was added under the appropriate tables. The value of *p* < .05 was regarded as statistically significant.

## 3 RESULTS

### 3.1 Comparison of the study groups

The study group consisted of three subgroups (Table 1). The comparison of age of the studied groups—control subjects aged 14.7 (11.3–17.7), patients with diabetes aged 16 (10.9–17.8) and DM1 + AIT patients aged 15.2 (11.1–18.0) years—did not show significant differences. The diabetes duration was 7 (1.7–12.9) and 8.7 (2.6–14.4) years and age at onset of disease was 9.3 (2.5–13.5) and 6.6 (1.2–12.2) years, respectively.

Serum concentrations of total cholesterol, low density lipoproteins (LDL) cholesterol and HbA1C were higher in DM1 + AIT group in comparison to healthy controls (*p* = .004, *p* = .02 and *p* < .0001; respectively). Serum concentrations of total cholesterol and HbA1C were also higher in DM1 group than in healthy controls (*p* = .048 and *p* < .0001). No other differences between groups were found (Table 1).

The comparison of all studied groups regarding carotid pulsatility (CCA_PI) and carotid resistance indices (CCA_RI) showed a significant difference between children with type 1 diabetes and autoimmune thyroiditis (DM1 + AIT) and healthy controls only. CCA_PI and CCA_RI were lower in DM1 + AIT than in healthy controls (*p* = .025 and *p* = .02, respectively; Table 2).

**Table 2:** Pulsatility indices and ABI in studied subgroups

Characteristics	Healthy control (C) *N* = 29	Diabetes mellitus t.1 (DM1), *N* = 19	Diabetes mellitus t.1 and autoimmune thyroiditis (DM1 + AIT), *N* = 19	*p*	p between groups		
					DM1 versus C	DM1 + AIT versus C	DM1 versus DM1 + AIT
CCA_PI	2.04 (1.43–3.12)	1.95 (1.42–2.42)	1.8 (1.35–2.39)	**.03**	.51	^a^ **.25**	.75
CCA_RI	0.77 (0.66–0.84)	0.74 (0.67–0.82)	0.73 (0.65–0.78)	**.02**	.30	^a^ **.02**	.95
brachial_PI	2.35 (1.96–3.06)	2.44 (1.97–3.22)	2.31 (1.84–2.96)	.40	–	–	–
thigh_PI	2.18 (1.97–2.71)	2.31 (2.01–2.93)	2.21 (1.96–2.57)	.51	–	–	–
above_knee_PI	2.42 (1.92–2.97)	2.24 (2.01–3.48)	2.26 (1.8–2.63)	.12	–	–	–
below_knee_PI	2.48 (2.02–3.18)	2.64 (2.21–3.55)	2.59 (2.22–3.69)	.12	–	–	–
ankle_PI	2.61 (2.23–3.92)	2.51 (2.19–3.39)	2.42 (1.96–3.06)	.29	–	–	–
ABI	1.03 (0.66–1.68)	1.09 (0.97–1.31)	1.09 (0.93–1.34)	.12	–	–	–

Note

Values are presented as median (minimum; maximum). The value of *p* < .05 was regarded as statistically significant (in bold).

^a^ The level of significance of comparison between the groups according to post hoc Fisher's test.

### 3.2 Comparison of arterial indices

The comparison of the pulsatility indices in the studied muscular arteries and ABI did not reveal any significant differences between the studied groups (Table 2).

In control subjects, the CCA_RI significantly correlated with total cholesterol and LDL cholesterol levels (*r* = 0.39, *p* = .04; *r* = 0.39, *p* = .04, respectively).

The analysis revealed that in the studied groups correlation between PI and age was not the same in all examined arteries. In the control group, a significant correlation with age was found for common carotid and brachial arteries (*r* = 0.38, *p* = .045; *r* = 0.49, *p* = .007, respectively). In the DM1 group, a significant correlation with age was found for above_knee_PI, below_knee_PI and ankle_PI (*r* = 0.49, *p* = .03; *r* = 0.52, *p* = .03 and *r* = 0.66, *p* = .002, respectively). In DM1 + AIT group significant correlation with age was found for below_knee_PI and ankle_PI (*r* = 0.47, *p* = .04 and *r* = 0.59, *p* = .01, respectively).

Age of diabetes onset, duration of the disease, insulin dose, treatment with pump, and HbA1C level showed no correlation with PIs and RI in the DM1 and DM1 + AIT groups.

**Figure 2:**
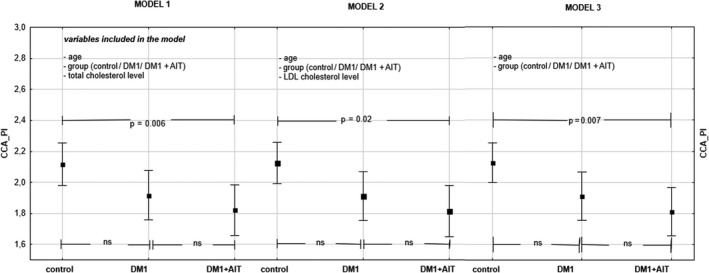
The comparison of CCA_PI between groups. (Model 1: adjusted means: age ‐ 15 years, total cholesterol ‐ 178 mg%; Model 2: adjusted means: age‐ 15 years, cholesterol LDL 103 mg%; Model 3: adjusted means: age‐ 15 years)

Multivariate models include: age, the presence of comorbidities, total cholesterol (model 1), and LDL cholesterol (model 2) as confounders. The analysis results of both models have not indicated the impact of total or LDL cholesterol on the common carotid pulsatility index. The age of the respondents and comorbidities present impacted CCA_PI significantly, that has been shown in the Model 3 defined with the exception of lipids level (Table 3, Figure 2). Power of the study (for α = 0.05) is 0.69.

**Table 3:** General linear model of CCA_PI confounders

Effect	SS	*df*	MS	*F*	*p*
Model 1 (Figure 2)					
Constant	1.225	1	1.225	10.584	**.002**
Age	0.598	1	0.598	5.166	**.027**
Total cholesterol	0.019	1	0.019	0.164	.687
Group (C/ DM1/ DM1 + AIT)	0.860	2	0.430	3.716	**.030**
Error	7.175	62	0.116		
Model 2 (Figure 2)					
Constant	1.356	1	1.356	11.69	**.001**
Age	0.626	1	0.626	5.39	**.023**
LDL cholesterol	0.001	1	0.001	0.01	.932
Group (C/ DM1/ DM1 + AIT)	1.013	2	0.507	4.37	**.017**
Error	7.193	62	0.116		
Model 3 (Figure 2)					
Constant	1.909	1	1.909	16.72	**<.001**
Age	0.633	1	0.633	5.54	**.022**
Group (C/ DM1/ DM1 + AIT)	1.22	1	1.610	5.34	**.007**
Error	7.194	63	0.114		

The value of *p* < .05 was regarded as statistically significant (in bold).

## 4 DISCUSSION

In our study we have shown changes in the properties of large arteries in a group of children with noncomplicated type 1 diabetes with coexisting autoimmune thyroiditis. The measurements were comparable between children with diabetes without AIT and healthy subjects. This effect was independent of patient age. We have We also demonstrated that in patients with diabetes and coexisting AIT, early macroangiopathy is more likely to affect elastic arteries, such as the common carotid artery, rather than the muscular blood vessels in the limbs.

Despite that PI is widely used to evaluate macrovasculature and may reflect its early functional impairment, no data on the influence of AIT on PI in children have been presented in literature. The discussion of obtained results regarding macrovascular system is limited due to the lack of other studies on properties of large arteries in corresponding pediatric cohorts. The literature review revealed only one study performed on adolescents and young adults with diabetes, which showed that the presence of additional autoimmune disease does not significantly affect the value of carotid intima‐media thickness (IMT; Klonowska et al., 2016). However, the functional effects of AIT have not been studied in their research. The results of studies based on adults with Hashimoto's thyroiditis without type 1 diabetes are inconsistent (Ciccone et al., 2010; Nyirenda et al., 2005; Stamatelopoulos et al., 2009; Topaloglu, Gokay, & Kucukler, 2013; Zöller, Li, Sundquist, & Sundquist, 2012a, 2012b). Stamatelopoulos et al. showed that the stiffness of arteries, but not the carotid IMT, was greater in euthyroid patients with Hashimoto's thyroiditis compared to controls (Stamatelopoulos et al., 2009). Conversely, other authors have shown that overweight and obese patients with Hashimoto's thyroiditis at euthyroid stage, as well as premenopausal women with euthyroid Hashimoto thyroiditis had thicker IMT compared with those without autoimmune disease (Ciccone et al., 2010; Topaloglu et al., 2013). Moreover, patients with Hashimoto thyroiditis aged over 50 years had higher rates of hospitalization for cardiovascular disease, as well as higher rates of coronary heart disease and stroke when compared to control groups (Nyirenda et al., 2005).

PI is recognized as a measure of elastic properties of large arteries according to some studies (Babcock et al., 2015; Eberth et al., 2009; Wohlfahrt et al., 2014). Our data have shown that the examined children with diabetes mellitus, both with and without autoimmune thyroiditis, did not have any structural changes in large arteries yet. Both the pulsatility index of elastic and muscular arteries as well as the ankle‐brachial index are commonly used to characterize large arteries. In the groups of children that we have examined, we show differences in pulsatility indices in the common carotid arteries only. The significant differences found in the pulsatility index of elastic, but not muscular arteries, present between the DM1 + AIT group when compared to the control group, may indicate that the elastic arteries are more susceptible to DM1 in the presence of AIT compared to muscular arteries.

Diabetes enhances arterial stiffness through a variety of mechanisms, such as a decreased availability of nitric oxide, leading to endothelial dysfunction, and a persistent inflammatory state (Oliveira et al., 2013). Diabetes is also associated with impairment of the connective tissue composition in the arterial wall, namely collagen cross‐linking and enhanced glycation of collagen (Cameron & Cruickshank, 2007; Shirwany & Zou, 2010).

All arteries increase in stiffness with age. This is further augmented by other factors, such as an increase in blood pressure, dyslipidemia or persistent hyperglycemia. The more proximal, more elastic arteries tend to be significantly more affected by aging than the more distal, muscular arteries. This is most likely due to the differences in proportions of smooth muscles, collagen and elastin (Cameron, Bulpitt, Pinto, & Rajkumar, 2003; Shirwany & Zou, 2010). The highly elastic arteries with their much thicker elastic media, are likely more sensitive to the effects of collagen cross‐linking, glycation, and the effects of decreased nitric oxide availability. The significant differences found in the pulsatility index of elastic, but not muscular arteries, present between the DM1 + AIT group when compared to the control group, also support the idea that the elastic arteries are more susceptible to factors resulting in increased vascular stiffness, such as hyperglycemia, disorders of lipid metabolism and persistent inflammation.

Previously, we have demonstrated how skin microcirculation deteriorates in patients with diabetes and autoimmune thyroiditis (Hoffmann et al., 2019). The significantly lower pulsatility index in elastic arteries found in our current study further supports the idea that these two conditions have a negative impact on the cardiovascular system. To the best of our knowledge, this is a new finding not previously published in other studies.

In healthy subjects PI is an age‐dependent parameter. This fact was described by Schoning et al (Schöning & Hartig, 1998; Schöning, Walter, & Scheel, 1994) in their studies applying ultrasound examination of PI for common carotid artery in healthy children, adolescents and adults. They showed that PI changes from value 1.73 ± 0.31 for children under 10 years of age (Schöning & Hartig, 1998) through 1.97 ± 0.42 in children and adolescents over 10 years of age (Schöning & Hartig, 1998) to 1.72 ± 0.5 for adults (Schöning et al., 1994). The significant differences of PI between groups are shown. It is worth noticing that according to Shoning et al, pulsatility index at common carotid in adults artery significantly decreases with age (*r* = −0.52 *p* = .001; Schöning et al., 1994). The study data analysis revealed that in control groups age was significantly correlated with both CCA_PI and brachial_PI. Such age‐dependence was not found for DM1 and DM1 + AIT groups.

Dyslipidemia is an important problem in both DM1 and AIT patients. In type 1 diabetic patients, dyslipidemia is an important cause of morbidity and mortality as it leads to both micro and macrovascular complications through arterial stiffening and occlusion (Gylling, Tuominen, Koivisto, & Miettinen, 2004). It has been suggested that the low expression of ABC G/5 G/8 genes in those patients results in high absorption of cholesterol and sterols in general, while the synthesis of cholesterol is decreased (Miettinen, Gylling, Tuominen, Simonen, & Koivisto, 2004). Autoimmune thyroiditis also has significant effects on lipoprotein metabolism. Subclinical hypothyroidism can slow down metabolic pathways of cholesterol uptake, synthesis and secretion, with the distinctive feature being reduced synthesis of hepatic LDL receptors (Vukovic et al., 2019). Additionally, the chronic inflammation accompanying any autoimmune disease process also has detrimental effects on vascular health (Bojanin, Milenkovic, & Vekic, 2018). Thus, DM1 and HIT both significantly contribute to dyslipidemia through their disease‐specific mechanisms as well as the global effect of chronic inflammation and, as such, it could be expected that lipid levels would be higher in patients with both conditions rather than DM1 alone. This is supported by the findings of Bojanin et al (2018), who studied the effects of coexisting autoimmune diseases, namely celiac disease (CD) and autoimmune thyroiditis (AIT) on serum lipids and lipoprotein subclasses profile in pediatric patients with DM1. Their results showed that coexisting autoimmune diseases had a significant impact on lipid profiles of this patient population. Patients with DM1 + CD and DM1 + AIT had significantly lower HDL‐C concentrations when compared to the DM1 only group. Additionally, patients with high LDL‐C and low HDL‐C were more prevalent in the DM1 + CD and DM1 + AIT groups. Similar patterns were found by Korzeniowska et al, who demonstrated that patients with DM1 + AIT had lower HDL‐cholesterol levels and higher triglyceride levels when compared to patients with DM1 alone (Durante & Bronzato, 2015). In our study, the lipid profile analysis revealed that total cholesterol level was higher both in DM1 + AIT and DM1 groups than in control group. A significant difference was found in LDL cholesterol between DM1 + AIT and control groups, whereas study groups did not differ according to HDL level. According to present recommendations in children LDL over 100 mg% is considered abnormal, over 130 mg% is the level to start lipid‐lowering therapy (Graves & Donaghue, 2019). Using these values as cut‐off points, we found hyperlipidemia in 53% of the patients in the DM1 group and in 68% of the patients in the DM1 + AIT group. Additionally, 21% of the DM1 patients and 32% of the DM1 + AIT patients had indications for initiation of lipid‐lowering therapy. Similarly, our study, conducted on children over 10 years old, has shown that the DM1 + AIT group, but not the DM1 group, had significantly lower PI values for the common carotid artery in comparison to healthy children. The values of pulsatility index in the group examined by us are similar to those published by Schoning et al. Elevated lipid levels are a known risk factor for vascular stiffness and vessel aging. They have a significant impact on arterial properties, which is reflected by the lower PI values. This was further supported by the negative correlation between age and PI demonstrated by Schoning et al (Schöning et al., 1994). Our comparison of studied groups with regards to total cholesterol or LDL cholesterol levels and age showed that CCA_PI is significantly determined by the coexistence of autoimmune thyroid disease and age.

Therefore, it seems reasonable to hypothesize that in patients with diabetes, the coexistence of autoimmune thyroid disease causes faster vascular aging.

The limitation of our study may be the small numbers of patients in the studied subgroups, which could potentially influence the power of study. Evidently, our results should be confirmed with a larger cohort. Thus, the presented results should be regarded as a pilot study. In our opinion, the presented results provide additional data which are important in understanding the meaning and significance of pulsatility index in the pediatric population.

In conclusion, our study showed that the presence of autoimmune thyroiditis affects the function of elastic but not muscular arteries in children with type 1 diabetes mellitus independent of lipid levels. This may serve as a valuable addition to the discussion on the need for introducing lipid‐lowering therapy in children with type 1 diabetes and coexisting autoimmune diseases.

## 5 ETHICS APPROVAL STATEMENT AND PATIENT CONSENT STATEMENT

All procedures performed in studies involving human participants were in accordance with the ethical standards of Ethics Committee in Gdansk, Poland and with the 1964 Helsinki declaration and its later amendments or comparable ethical standards. The study protocol was approved by the Medical Ethics Committee of the Medical University of Gdansk (NKBBN/277/2014; NKBBN/277‐512/2016). Upon entering the study participants' parents gave informed consent. Parents were present during the tests.

## 6 DATA SHARING NOTICE

The data used for the study can be shared upon personal contact with the Authors.

## CONFLICTS OF INTEREST

The author(s) declare no potential conflicts of interest with respect to the research, authorship, and/or publication of this article.

## AUTHOR CONTRIBUTIONS

J.N.G. examined the patients, designed the study, performed the analysis, wrote the manuscript, and contributed to the discussion. M.W. examined the patients, designed the study and wrote the manuscript. G.M.K. contributed to the discussion, and wrote the manuscript. Z.Z. contributed to the discussion. M.M. recruited patients, and contributed to the discussion. L.B. is the guarantor of this work, and took full responsibility for the work as a whole, designed the study, performed the analysis, wrote the and manuscript, decided to submit and publish the manuscript.
